# ASO Author Reflections: Is Survival Sufficient? There’s More to Address for the Breast Cancer Surgeon

**DOI:** 10.1245/s10434-020-09350-4

**Published:** 2020-11-17

**Authors:** Cary S. Kaufman

**Affiliations:** 1 Department of Surgery, University of Washington, Seattle, WA USA; 2 Bellingham Regional Breast Center, Bellingham, WA USA

## PAST

Many readers may not have heard of Rose Kushner, one of the first lay advocates leading the effort to change the conventional one-step biopsy/radical mastectomy procedure to a two-step procedure, with diagnostic biopsy preceding mastectomy.^1^ This was long before breast conservation or the use of needle biopsies for diagnosis, at a time when the 5-year survival rate was 75%. Currently, the 5-year survival rate for breast cancer has reached over 91%, mastectomy for early breast cancer has largely been replaced by breast-conserving surgery, and neoadjuvant treatment often results in pathologic complete response. Because most patients survive this disease, it is incumbent upon surgeons to complement the treatment of their patients by lessening the side effects from such treatment. One of the recognized and unfortunate results of breast-conserving surgery has been cosmetically “less than ideal” results.^2^ Surgeons have traditionally been taught to leave seromas in place because the patients appeared to have normal contour during short-term follow-up assessment. Only after the patient returned from radiation therapy did the lumpectomy site look sunken and retracted. However, a few surgeons recognized that the aesthetic result could be better. They advised altering the management of the lumpectomy bed by closing the dead space and better focusing radiation therapy. Closing the bed avoided retraction of the lumpectomy site, whereas improvement in radiation therapy planning decreased the overall extent of radiation injury.

## PRESENT

In 2012, Dr. Gail Lebovic received Food and Drug Administration (FDA) approval to use a three-dimensional (3D) bioabsorbable target for radiation therapy planning and initiated the registry study that we report.^3^ That year, the BioZorb® was used first in New Zealand, followed by the United States, which provided more benefits than initially expected.^4^,^5^ The main purpose of the device was to better target radiation therapy to the lumpectomy bed due to several noted problems with clip placement.^6^ One advantage, among others noted in our report, was that it allowed radiation therapists to avoid treating inadvertent seromas and oncoplastic surgical dissection sites not related to the cancer excision but a byproduct of reconstructive lumpectomy. An unexpected benefit of the device was that in providing this structure for lumpectomy site identification and targeting, it also maintained the shape and contour of the breast while the normal healing (and scarring) process occurred. In our study, good to excellent cosmetic results were noted by the vast majority of patients and doctors. Preventing the retraction that occurred in the past after radiation therapy was an important finding with use of the BioZorb®. The multiple benefits of a 3D bioabsorbable tissue marker are underscored by its current widespread use in the United States.

## FUTURE

This study provided evidence that the combination of breast-conserving surgery with BioZorb® placement has favorable cosmetic outcomes and an acceptable safety profile. In the future, early diagnosis with 3D screening mammography will bring many patients to the surgeon with breast cancer identified early in the evolution of disease. Many of these women will be treated with breast-conserving surgery and have long-term survival. The surgeon of tomorrow will be responsible for more than just the oncologic outcome. The surgeon also will be expected to weigh the side effects of their treatment more carefully, enhance the cosmetic outcome, evaluate the long-term effects of radiation, consider the issues of physical mobility, and assess genetic risk and overall emotional health. In our opinion, novel technologies such as the BioZorb® will be an invaluable addition to the comprehensive care provided by the surgeon of tomorrow.
